# Tryptophan-Degrading Enzymes in Tumoral Immune Resistance

**DOI:** 10.3389/fimmu.2015.00034

**Published:** 2015-02-03

**Authors:** Nicolas van Baren, Benoît J. Van den Eynde

**Affiliations:** ^1^Ludwig Institute for Cancer Research, Brussels, Belgium; ^2^Walloon Excellence in Life Sciences and Biotechnology (WELBIO), Brussels, Belgium; ^3^de Duve Institute, Université catholique de Louvain, Brussels, Belgium

**Keywords:** indoleamine 2,3-dioxygenase, tryptophan-2,3-dioxygenase, dendritic cells, tumor, immunosuppression, tryptophan, adaptive resistance

## Abstract

Tryptophan is required for T lymphocyte effector functions. Its degradation is one of the mechanisms selected by tumors to resist immune destruction. Two enzymes, tryptophan-2,3-dioxygenase and indoleamine 2,3-dioxygenase 1, control tryptophan degradation through the kynurenine pathway. A third protein, indoleamine 2,3-dioxygenase 2, was identified more recently. All three enzymes were reported to be expressed in tumors, and are candidate targets for pharmacological inhibition aimed at restoring effective anti-tumoral immunity. In this review, we compare these three enzymes in terms of structure, activity, regulation, and expression in healthy and cancerous tissues, in order to appreciate their relevance to tumoral immune resistance.

## Introduction

In the last decade, tryptophan catabolism has emerged as a powerful mechanism of peripheral immune tolerance, contributing to maintain homeostasis by preventing autoimmunity or immunopathology that would result from uncontrolled and overreacting immune responses. This is achieved through the action of enzymes catalyzing the first and rate-limiting step of tryptophan degradation along the kynurenine pathway, including indoleamine 2,3-dioxygenase 1 (IDO1) and tryptophan-2,3-dioxygenase (TDO). As a result, tryptophan is locally depleted while tryptophan catabolites accumulate, including kynurenine and its derivatives, depending on the presence of downstream enzymes in the kynurenine pathway. Although IDO1 and TDO are located in the cytosol, the metabolic modifications they induce extend to the extracellular microenvironment because tryptophan and kynurenine derivatives readily cross the plasma membrane through specific transporters (1–3). These metabolic modifications result in a local microenvironment becoming profoundly immunosuppressive, as a result of various mechanisms whose respective role remains incompletely characterized. A first mechanism is based on tryptophan depletion: T lymphocytes are extremely sensitive to tryptophan shortage, which causes their arrest in the G1 phase of the cell cycle (4). This is due, at least partly, to the induction of an integrated stress response triggered by GCN2, a stress kinase that is activated by elevations in uncharged tRNAs (5). Tryptophan shortage can also stop T-cell proliferation through inactivation of the mTOR pathway (6). A second mechanism depends on the accumulation of tryptophan catabolites: some of them, such as 3-hydroxyanthranilic and quinolinic acids, can induce T-cell apoptosis (7, 8), while other kynurenine derivatives can induce the differentiation of regulatory T-cells (9), possibly through activation of the aryl hydrocarbon receptor (AhR) (10).

One of the key hallmarks of cancer is the ability to evade immune destruction (11). To do so, tumors often hijack one or several of these tryptophan-catabolizing enzymes endowed with immunosuppressive properties. The best studied enzyme in this respect is IDO1. In 2003, our group showed for the first time that IDO1 was expressed in human tumors, and that, in a mouse tumor model, IDO1 protected tumors against immune rejection, an effect that could be reversed by pharmacological IDO1 blockade (12). Numerous subsequent studies have confirmed these findings and demonstrated, in mouse models, the benefit of IDO1 inhibitors, either alone or in combination with chemotherapy (13, 14). Several IDO1 inhibitors currently undergo clinical development. More recent work from our group demonstrated that also TDO was expressed in human tumors and showed a similar tumor-protective effect against immune rejection (15). Tumor rejection was restored by pharmacological TDO inhibition, making TDO a second attractive target for cancer therapy. More recently, indoleamine 2,3-dioxygenase 2 (IDO2), a third enzyme potentially involved in tryptophan catabolism, was identified through its high homology with IDO1 (16, 17). Its precise activity and its involvement in tumoral immune resistance have not been clearly established.

Tryptophan-2,3-dioxygenase, IDO1, and possibly IDO2 catalyze the same reaction of oxidative breakdown of the indole group of tryptophan. However, they differ in a number of important aspects such as structure, activity, regulation, and tissular expression. In this review, we discuss the aspects of these enzymes that are relevant to their potential immunosuppressive role in the context of tumoral immune resistance, in view of recent data highlighting their expression profile in normal and cancerous human tissues.

## Tryptophan-2,3-Dioxygenase

Tryptophan-2,3-dioxygenase is a 167 kDa tetrameric heme-containing enzyme with constitutive and specific l-tryptophan-catabolizing activity. It is selectively expressed in the liver, where it regulates the levels of blood tryptophan. Consistent with this regulatory function, human TDO has a *K_m_* of 190 μM for l-tryptophan, which allows it to efficiently degrade this amino acid at concentrations above its physiological level (around 80 μM) (18). As the main enzyme responsible for the metabolism of dietary tryptophan, TDO is positively regulated by tryptophan, which dramatically increases TDO expression and/or activity when present in the blood at supraphysiological concentrations (19). These features appear optimized to fulfill the main function of TDO, i.e., to maintain tryptophan homeostasis. This principal function of TDO was confirmed by the observation of a 10-fold increased tryptophan level in the blood of TDO-knockout as compared to wild-type mice (20).

**Figure 1:**
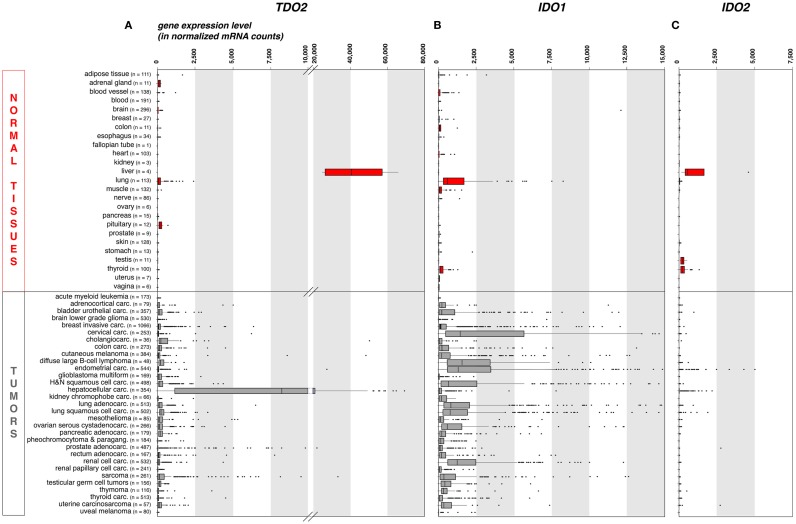
***TDO2, IDO1*, and *IDO2* gene expression in common human normal and tumoral tissues (panels A, B and C, respectively)**. We used publicly available whole transcriptome data to assess the expression of these three genes in large series of human normal and cancerous tissues. We retrieved raw mRNA counts from the Genome Tissue Expression project (GTEx, see text footnote 1) and The Cancer Genome Atlas (TCGA, see text footnote 2) databases, respectively, normalized the values according to the DESeq approach (23), and represented the results as boxplot graphs for each normal tissue and tumor type, using the R statistical software and the Bioconductor package. The DESeq normalization approach allows to correct the data for the sequencing depth, which affects the number of aligned reads to the gene. It (i) computes the geometric mean for each gene; (ii) divides raw counts for each gene by the corresponding geometric mean; (iii) computes for each sample the median of the obtained ratios; and (iv) divides each gene count by the computed median for the sample. The vertical bars in each box represent, from left to right, the first quartile, median, and third quartile of the indicated sample population. The left and right edge of the horizontal line represents the minimum and maximum values, respectively, after exclusion of the outliers, displayed as individual dots. Several (*n* = 37) IDO1 outlier values >15,000 have been omitted for the clarity and concision of the graphical display. This approach provides a robust mean to assess the expression of specific genes in the context of malignant diseases, because RNA-Seq data are more precise and have less background than microarray data, and because large series of highly controlled data of various sample types are available from public databases. Carc, carcinoma; H&N, head-and-neck; Paragang, paraganglioma.

As compared to liver, the expression of TDO in other normal tissues is negligible. This is illustrated on Figure 1A, where we compiled whole transcriptome data from the Genome Tissue Expression project (GTEx)^1^ (21). In contrast, a number of human tumors express significant levels of TDO. Figure 1A also shows a compilation of *TDO2* expression data in 9,169 human tumors, retrieved from whole transcriptome data from The Cancer Genome Atlas (TCGA)^2^ (22). Importantly, we used the same approach (DESeq) (23) to normalize the RNA-Seq data from normal and tumoral tissues, so as to allow comparison of expression levels, despite the distinct origins of the two data sets.

As expected because of its expression in normal liver, *TDO2* is expressed at high levels in hepatocarcinoma (Figure 1A). It is also expressed in many other tumor types, although at weaker levels and only in a fraction of the samples. These data are in line with published RT-qPCR data obtained in a smaller series of human tumor samples (15), and corroborate reported findings of constitutive TDO expression in a number of established human tumor cell lines of various histologies, including glioblastoma, colorectal carcinoma, head-and-neck carcinoma, and gallbladder carcinoma (15, 24). The precise cell type(s) expressing TDO in liver and tumors remain(s) to be identified, awaiting the availability of a reliable validated antibody.

Given the immunosuppressive effect of tryptophan catabolism, this TDO expression in human tumors prompted us to evaluate whether TDO favors tumor growth by promoting resistance to immune rejection. Due to the lack of mouse tumors naturally expressing TDO, we resorted to TDO-transfected tumor lines, and observed that TDO-positive P815 tumors were no longer rejected by mice immunized against P1A, a MAGE-type tumor antigen naturally expressed by this mastocytoma (15). Moreover, based on a previously published scaffold (25), we developed a new TDO inhibitor with a better bioavailability after oral administration (26), and observed that mice treated with this compound recovered their ability to reject TDO-expressing tumors (15). The treatment was not associated with any noticeable toxicity nor elevation of liver enzymes. These results made the proof of concept for the use of TDO inhibitors as immune modulators for cancer therapy. In parallel, another group provided evidence for a tumor cell autonomous effect of TDO expression in glioblastoma, promoting tumor progression through AhR activation by tryptophan catabolites, resulting in increased tumor cell survival and motility, and reduced anti-tumor immune responses (24). The notion that the effects of TDO on tumor growth and anti-tumor immunity would be primarily mediated by tryptophan catabolites – as opposed to tryptophan depletion – fits with the high *K_m_* of TDO for tryptophan, which makes this enzyme more apt at producing significant amounts of tryptophan catabolites than at depleting tryptophan down to the submicromolar levels needed to impair T-cells.

Besides IDO, these results establish TDO as another immunosuppressive enzyme involved in tumor progression, and make it a promising drug discovery target. In addition, the high expression of TDO in the liver raises interesting questions regarding its potential immunosuppressive role in this organ. Liver is known as an immune tolerant organ: as opposed to other transplanted tissues, HLA-mismatched liver allografts are usually well tolerated in humans and require less immunosuppressive therapy (27). It will be interesting to evaluate whether TDO plays a role in this phenomenon and to determine whether TDO inhibition could alter the course of liver infections, primary liver tumors, or liver metastases. In line with the notion of a protective effect of TDO against excessive liver immunity and/or inflammation, TDO was recently found to play a key role in the protection against endotoxic shock: TDO-knockout mice died after injection of a LPS dose that was only sublethal in wild-type animals (28). Interestingly, AhR-knockout mice were equally sensitive to sublethal LPS, in line with the notion that a tryptophan catabolite produced by TDO – possibly kynurenine – was responsible for the protective effect by activating AhR.

## Indoleamine 2,3-Dioxygenase 1

Indoleamine 2,3-Dioxygenase has been known for more than four decades as an intracellular tryptophan-degrading enzyme whose expression is strongly induced by IFNγ in most cells. Although IDO1 catalyzes the same reaction as TDO, the two enzymes are unrelated in terms of primary structure and also differ in quaternary structure. Both are heme-containing enzymes requiring a reduced iron atom in the catalytic site, but IDO1 functions as a monomer while TDO is a tetramer of four heme-containing subunits (29). The enzymes further differ in terms of expression profile (see below) and substrate specificity, with IDO1 acting on a larger variety of substrates of the indoleamine family. Lastly, IDO1 has a much lower *K_m_* for tryptophan (about 20 μM), making this enzyme able to significantly deplete tryptophan down to the submicromolar range (30). As such IDO1 was long considered as one of the effector molecules of IFNγ, acting to limit the growth of intracellular pathogens by depriving tryptophan. In the late 90s, the immunosuppressive function of IDO1 was discovered in mice by studying the placenta, which constitutively expresses high levels of IDO1: tryptophan catabolism by placental IDO1 was found instrumental in protecting the fetus from maternal immune rejection (31). Subsequent studies amply confirmed this immunosuppressive role of IDO1, first in dendritic cells (DC), where IDO1 can be induced by a number of stimuli that drive DC toward a tolerogenic program (32, 33), but also in tumors, which often express IDO1 and thereby resist immune rejection (12). These findings shed a new light on the function of IFNγ-induced IDO1: more than an effector mechanism of IFNγ, IDO1 now appears as part of a retrocontrol mechanism responsible for the termination of immune responses and the prevention of immunopathology, which would result from overreacting responses. Numerous preclinical studies have subsequently documented the therapeutic potential of IDO1 inhibitors in cancer therapy, either alone or in combination with chemotherapy or immunotherapy (13, 14, 34). Inhibition of the IDO1 pathway therefore represents a promising therapeutic approach, and clinical trials evaluating the first IDO1 inhibitors have started. IDO1 inhibition does not cause obvious toxicity in mouse models, and IDO1-knockout mice do not display abnormal phenotypic features (35, 36), suggesting that the approach is safe. As expected, IDO1–KO mice displayed increased sensitivity to the induction of inflammatory and autoimmune reactions (on-target effects) (37). They also displayed pericardiac calcifications, but this was observed in only 30% of females of only one strain of mice (37). Because the IDO1 expression profile differs between mouse and man, a better prediction of potential side effects of IDO1 inhibition requires a careful evaluation of IDO1 expression in human tissues. We recently developed and validated a new highly specific monoclonal antibody against IDO1, and used it to perform an extensive profiling of IDO1 expression in normal and tumoral tissues by immunohistochemistry (38). We discuss the main findings of this work in the following paragraphs.

### IDO1 expression in human non-cancerous tissues

In normal human tissues, the IDO1 protein was observed in mature DC located in lymphoid organs, in some epithelial cells of the female genital tract, as well as in endothelial cells of term placenta and, surprisingly, lung parenchyma (38). These data are consistent with the expression profile of the *IDO1* transcript that we obtained from the GTEx database using the same approach as for *TDO2* (Figure 1B). The *IDO1* gene is weakly or not expressed in most normal tissues, with the noticeable exception of lung. Note that placenta and secondary lymphoid organs, which also contain IDO1-expressing cells, were not represented in the GTEx data set that we used.

#### Healthy non-lymphoid tissues

Indoleamine 2,3-Dioxygenase 1 is constitutively expressed in human and mouse placenta. In the latter, its involvement in fetal protection against maternal immune rejection has been demonstrated experimentally (31). This function cannot be readily extrapolated to human pregnancies, because the cell types that express IDO1 differ between the two species. In the mouse, IDO1 expression was observed in trophoblast cells (35, 39) whereas in human placenta it was found in endothelial cells (38, 40). The functional consequences of endothelial IDO1 expression in terms of tryptophan degradation and immune protection are not known. The same applies to lung endothelium. It is hard to consider that tryptophan catabolism by cells exposed to the blood flow can impose an immunosuppressive flavor to the microenvironment. Therefore, a cell-intrinsic function is more likely. In this regard, it is interesting to note that the density of the pulmonary vasculature was reported to be reduced in IDO1-deficient as compared to wild-type mice, suggesting a role for IDO1 in supporting lung vascular development (41). In mice, inflammation-induced IDO1 expression in endothelial cells was also reported to induce vasodilation and contribute to reduced blood pressure during severe inflammation (42). Apart from IDO1 vascular expression, lung and placenta have another exclusive feature in common. Both are respiratory organs, in which the oxygen and carbon dioxide gradients are inverted as compared to peripheral organs. Whether there is a link between these two features, and whether these gradients affect the redox status of IDO1, which controls its enzymatic activity, is not known.

Constitutive expression of the IDO1 protein has also been reported in epithelial cells from the female genital tract (38, 43). It has been hypothesized that this expression helps fight genital infections through local depletion of tryptophan, required for pathogen growth. In the mouse, one of the tissues with the highest expression of IDO1 is the epididymis. This specific tissular expression was not observed in man (38).

#### Healthy lymphoid tissues

Interstitial cells expressing IDO1 were observed in human lymphoid tissues, including lymph nodes, spleen, tonsils, Peyers’s patches, the gut lamina propria, and the thymic medulla (38). These cells were further characterized in lymph nodes and lamina propria, and were identified as mature conventional DC. They all expressed maturation markers DC-LAMP and CD83, and lacked markers of other DC or myeloid subtypes such as CD1a, langerin, CD123, and CD163. In lymph nodes, about 50% of mature conventional DC expressed IDO1, while neither plasmacytoid DC (pDC) nor any other cell type did. This parallels the IDO1 induction observed *in vitro* during maturation of human monocyte-derived DC (MoDC), which happens during the terminal phase of the DC maturation program, and likely represents a negative feedback loop of retrocontrol of the immune response (38). Various maturation stimuli can induce IDO1 in MoDC, including LPS and the cocktail of cytokines [interleukin-(1)-beta, interleukin-6, tumor necrosing factor-alpha, and prostaglandin-E2 (PGE2)] commonly used to produce DC-based vaccines for clinical immunotherapy (44). Among the cytokines present in this maturation cocktail, PGE2 plays the key role for IDO1 induction (45). Interestingly, omitting PGE2 from the cytokine cocktail results in fully mature MoDC lacking IDO1 expression, which should be more efficient as a vaccine platform (38).

Elegant mechanistic studies have characterized the induction and function of IDO1 in murine DC, particularly in pDC (46, 47). These studies showed a key role for IDO1 in controlling tolerogenic properties of pDC, and uncovered a complex regulation integrating transcriptional induction of IDO1, proteasome-mediated IDO1 degradation triggered under inflammatory conditions by IL-6 and SOCS3, and IDO1-mediated signaling leading to long-term tolerance via transforming-growth factor beta production. It is unclear at the present time whether those findings also apply to human pDC, which do not express IDO1 (38, 48).

### IDO1 expression in tumors

#### At the tumor site

Tumors have diverted the immunosuppressive function of IDO1 to their own benefit in their continued efforts to resist immune rejection. A number of human tumor cell lines express IDO1 in a constitutive manner, and most other tumor lines start expressing IDO1 when exposed to IFNγ (12). In human tumor samples, IDO1 expression is commonly observed both at the RNA and protein levels (Figure 1B) (38, 49). Murine tumors expressing IDO1 are resistant to immunization-dependent rejection, a phenomenon that can be partially reverted by IDO1 inhibition (12). In human tumors, IDO1 expression usually correlates with a poor prognosis and is linked to a more aggressive tumor phenotype, a reduced tumor infiltrate, and an increased number of regulatory T-cells at the tumor site (49).

Our recent immunohistochemistry study analyzed 866 human tumors of 15 common types: about 56% expressed IDO1 (38). There was a remarkable match between the hierarchy of our IDO1 protein expression profile per tumor type and that of the corresponding mRNA retrieved from the TCGA database (Figure 1B). In both datasets, endometrial and cervical carcinomas emerged as the tumors with the highest and most frequent IDO1 expression, followed by kidney and lung carcinomas. The lowest values were in both cases observed in glioblastomas. Three distinct cellular expression patterns emerged, individually or in combination. IDO1 was expressed by tumor cells (20% of the samples), by interstitial cells in lymphocyte-rich areas in the tumor stroma (46% of the samples), or by endothelial cells (14% of the samples). Part of the IDO1 expression by tumor cells might result from an ongoing immune response involving T lymphocytes producing IFNγ. This is exemplified by cervical carcinoma, where IDO1-positive tumor cells were often located at the periphery of tumor nodules, which were surrounded by T lymphocytes. This is reminiscent of the expression profile observed for PD-L1, another protein involved in tumoral immune resistance, which is also induced by IFNγ and often observed in T-cell infiltrated tumors. This PD-L1 expression profile, indicative of an adaptive resistance mechanism, was found to predict clinical responses to PD1/PD-L1 blocking reagents (50–52). In a similar manner, IDO1 expression in inflamed tumors might also indicate an adaptive resistance mechanism. In line with this, IDO1 expression in human melanoma was found to correlate with T-cell infiltration (53). Moreover, and quite paradoxically, IDO1 belongs to the group of genes whose expression in tumors prior to immunotherapy is predictive of a better clinical response, along with T-cell specific genes and other IFNγ-induced genes (54). Here, the key predictive factor is most likely the presence of tumor-infiltrating lymphocytes (TILs) inside the tumor, and IDO1 is secondarily induced in response to IFNγ produced by those TILs. In our study mentioned above (38), the IDO1 expression that was often observed in the tumor stroma also likely resulted from adaptive resistance. Such a pattern was dominant, for example, in colorectal carcinomas.

In contrast, a subset of tumors expressed IDO1 within tumor cells in the absence of any inflammation. This is the case in endometrial carcinomas, which often contain IDO1-expressing tumor cells scattered within tumor nodules in the absence of obvious T-cell infiltration (38). Constitutive IDO1 expression is also observed in a number of human tumor lines (12), and is likely triggered by oncogenic events, whose characterization will be of great interest (55). Tumor-intrinsic constitutive IDO1 expression might contribute to tumoral immune resistance by preventing T-cell infiltration, a mechanism conceptually different from adaptive resistance, where IDO1 expression would represent a negative feedback mechanism induced by the T-cell response. Intriguingly, constitutive IDO1 expression has not been observed in murine tumors so far. Many murine tumors (with the notable exception of B16 melanoma) express IDO1 upon exposure to IFNγ, but none of them expresses IDO1 in the absence of IFNγ. Therefore, preclinical models commonly used to evaluate IDO1 inhibitors investigate IDO1-related adaptive resistance but not intrinsic resistance (13). The latter, which is relevant to the human situation, can only be evaluated using models based on murine tumors stably transfected with IDO1 (12, 56). Recent evidence in mouse models indicates that both tumor cells and host-derived cells contribute to IDO1-mediated immune resistance to anti-CTLA4 therapy (56).

The last pattern of IDO1 expression observed in human tumors, which is particularly striking in kidney cancer, is restricted to endothelial cells (38). As discussed above, the biological function of endothelial IDO1 remains to be defined. Intriguingly, endothelial IDO1 expression in kidney tumors was reported to be associated with a better prognosis, while in most other tumor types, IDO1 expression is associated with a worse clinical outcome (57). Of note, as opposed to many other tumor types, T-cell infiltration of kidney tumors is associated with a bad prognosis (58). Further studies will be required to understand those unexpected features of kidney tumors.

#### In tumor-draining lymph nodes

Several publications have reported increased proportions of IDO1-expressing DC in tumor-draining lymph nodes (TDLN) as compared to normal lymph nodes, mostly in mouse tumor models but also in human tumors, suggesting an important role of TDLNs in shaping tumoral immune tolerance (59–62). However, the recent study mentioned above, which used a carefully validated monoclonal antibody against human IDO1, did not confirm an increased proportion of IDO1-expressing DC in a series of 30 human TDLNs obtained from human melanomas and breast carcinomas: these TDLNs expressed IDO1 at the same level as normal lymph nodes (38). These results suggest that the IDO1 expression that is relevant to tumor immunosuppression is located at the tumor site rather than in TDLNs. This is in line with recent findings showing that the use of IDO1 inhibitors in a mouse model barely affected the priming of new anti-tumor T-cells, but strongly reactivated effector T-cells *in situ* at the tumor site (34).

## Indoleamine 2,3-Dioxygenase 2

Indoleamine 2,3-dioxygenase 2 was identified based on its structural homology with IDO1 (16, 17). The corresponding genes are highly homologous and located adjacent to each other on the same chromosome, suggesting that they resulted from gene duplication. Both proteins share 43% identity at the amino acid level, including conserved residues that are important for IDO1 enzymatic activity. IDO2 is less well characterized than IDO1. Even though IDO2 has been reported to have enzymatic l-tryptophan degradation activity *in vitro* (17, 63), its *K_m_* for this amino acid is much higher than that of IDO1 and TDO, around 6.8 and 12 mM for the human and mouse enzymes, respectively (30, 64). These values are more than a 100-fold higher than the physiological l-tryptophan concentrations, making it unlikely that IDO2 plays a direct role in the degradation of this amino acid. They rather suggest that IDO2 has a different natural substrate (63).

Constitutive expression of Ido2 mRNA and protein has been detected in mouse kidney, liver, epididymis and brain (16, 17, 65). Upregulation by inflammatory stimuli was observed *in vitro* in murine DCs and mesenchymal stem cells treated with IFNγ, and *in vivo* in brain following *Toxoplasma gondii* infection (17, 66–68). *Ido2*-knockout mice have a normal embryologic development and do not show major phenotypic abnormalities, but some aspects of their inflammatory and immune response to antigenic stimulation are reduced, including skin contact hypersensitivity reactions, generation of regulatory T-cells, and production of pro-inflammatory cytokines (69). *Ido2*-/- mice also display decreased joint inflammation in a mouse model of rheumatoid arthritis (70). These experimental observations are difficult to reconcile with the proposed model of IDO2 functioning as an immunosuppressive enzyme, and suggest a more subtle immunoregulatory role for IDO2, also in line with its genetic epistatic interaction with IDO1, suggested by the altered IDO2 splicing observed in IDO1-knockout mice (69).

In contrast with the mouse, little is known about IDO2 expression and function in humans. This assessment is rendered difficult by the lack of a strictly validated antibody and by the complexity of human *IDO2* transcription. The human *IDO2* gene contains two functional polymorphisms in its coding sequence. The R248W polymorphism drastically reduces the measured enzymatic activity of IDO2, and the Y359X polymorphism generates a truncated, enzymatically inactive protein. The high prevalence of these polymorphic alleles results in a non-functional IDO2 enzyme in up to 50% of persons (17). In addition, the *IDO2* gene generates at least five alternative transcripts, with only one encoding the full IDO1-related protein. IDO2 mRNA was detected by RT-PCR in liver, small intestine, spleen, placenta, thymus, lung, brain, kidney, and colon, but the full-length transcript only in placenta and brain (17). Expression of the *IDO2* gene was detected by RT-PCR in circulating myeloid DC and pDC and in *in vitro*-matured MoDC (48, 71). However, it is not known whether the detected IDO2 transcripts encoded a functional IDO2 protein, as the PCR primers were chosen to amplify exons 9 and 10, which are present in all 5 IDO2 transcripts. Using PCR conditions that amplify exons 4–9, which are only present in the full-length IDO2-encoding mRNA, we failed to detect IDO2 expression in *in vitro*-matured MoDC (38).

There are insufficient experimental arguments to support the view that IDO2 is expressed in human tumors. Transcription of *IDO2* was detected by RT-PCR in a high proportion of a small number of gastric, colon, and renal carcinoma samples, as well as in several tumor cell lines treated with IFNγ, but again using primers amplifying exons 9 and 10 (72). The IDO2 protein was detected by Western blot in three IFNγ-incubated tumor cell lines (73). However, despite the fact that one of these lines was derived from a patient homozygous for the Y359X polymorphism, which results in a truncated protein, the size of the corresponding IDO2 band on the immunoblot was identical to the two others, raising doubts about the specificity of the antibody used. In addition, it is unclear whether this antibody was directed against IDO2 or IDO1. In the same study, IDO2 was detected by immunohistochemistry in the tumor cells from 12 out of 12 pancreatic carcinoma samples, but not in normal pancreatic tissue, using an anti-IDO2 polyclonal antibody. Here also, the antibody stained tumor samples from Y359X homozygous patients. Its specificity controls were not provided.

Our whole transcriptome analysis retrieved from public RNA-Seq databases displayed in Figure 1C shows that, among healthy human tissues, only liver, testis, and thyroid express the *IDO2* gene, whereas the overwhelming majority of human tumor samples (>99%) are negative. Because these data do not take the complexity of the alternative splicing of IDO2 transcripts into consideration, we verified the absence of IDO2-matched RNA-Seq reads in 20 melanoma metastases sequenced in our laboratory (data not shown). We also confirmed the absence of detectable *IDO2* expression by RT-qPCR in 128 human tumor samples and 25 human tumor cell lines of various histological types using primers amplifying exons 4–9 (data not shown).

Altogether, the frequent occurrence of non-functional *IDO2* alleles in the population, the lack of experimental evidence for a biologically relevant tryptophan catalyzing activity of IDO2 and the absence of detectable IDO2 expression in most tumors make it highly unlikely that IDO2 plays a significant role in tryptophan-related tumoral resistance against immune rejection in humans. This is supported by the observed trend toward increased overall survival in patients with wild-type IDO2 compared to patients with heterozygous or homozygous polymorphisms that ablate IDO2 activity, in a recent study of brain metastatic patients receiving radiotherapy combined with chloroquine (74).

## Summary

Three enzymes with different features have been implicated in the first step of tryptophan degradation along the kynurenine pathway. TDO is a tetrameric enzyme expressed at a high level in the liver, and responsible for degrading dietary tryptophan and maintaining constant levels of blood tryptophan. Besides this physiological role, which is supported by a high *K_m_* and a positive regulation of TDO by tryptophan, TDO appears to have an immunoregulatory role, probably mediated mostly by tryptophan catabolites. In contrast, IDO1 is an unrelated monomeric enzyme, whose expression is mostly inducible in most tissues, and which plays a key role in immunoregulation and the retrocontrol of immune responses. Its low *K_m_* allows IDO1 to effectively deplete tryptophan in the local microenvironment and thereby impair T-cell mediated immune responses. Both IDO1 and – albeit less frequently – TDO are expressed in human tumors and appear to play a role in tumoral immune resistance, which warrants ongoing drug discovery efforts aimed at the clinical development of IDO and TDO inhibitors for cancer therapy. Much less is known about the role of IDO2, the third enzyme of the pathway. IDO2 appears to be expressed at a low level in the liver, testis and thyroid. It is not significantly expressed in human tumors. The tryptophan-degrading activity of IDO2 is particularly low, with a *K_m_* at least 100-fold higher than the physiological concentration of tryptophan. This, together with the frequent occurrence of polymorphisms affecting the assumed catalytic activity of human IDO2, makes it unlikely that IDO2 by itself plays a direct role in tryptophan catabolism, but rather supports another function for this protein.

## Conflict of Interest Statement

Benoît J. Van den Eynde is co-founder of and consultant for iTeos Therapeutics, a company involved in the development of IDO and TDO inhibitors. Nicolas van Baren declares that the research was conducted in the absence of any commercial or financial relationships that could be construed as a potential conflict of interest.

## Footnotes

^1^http://www.gtexportal.org

^2^http://cancergenome.nih.gov

## Abbreviations

DC, dendritic cells; IDO1, indoleamine 2,3-dioxygenase 1; IDO2M, indoleamine 2,3-dioxygenase 2; IFNγ, interferon-gamma; MoDC, monocyte-derived DC; TDO, tryptophan-2,3-dioxygenase.
